# Short- and Mid-term Outcomes of the Extravascular Implantable Cardioverter-defibrillator in Pediatric and Adolescent Patients: A Single-center Experience

**DOI:** 10.19102/icrm.2025.16113

**Published:** 2025-11-15

**Authors:** Nikola Dragisic, Emily R. Backes, Lindsey Malloy-Walton, Edo K. Bedzra, William J. Gibson, Philip M. Chang

**Affiliations:** 1 Ward Family Heart Center, Children’s Mercy Kansas City, Kansas City, MO, USA

**Keywords:** Aurora, EV ICD, outcomes, pediatric

## Abstract

Experience with the Aurora extravascular (EV) implantable cardioverter-defibrillator (ICD) (Medtronic, Minneapolis, MN, USA) remains limited among pediatric centers in contrast to its broader implantation in adults. We evaluated our single-center experience with the EV ICD as part of our standard ICD options for treatment of malignant ventricular arrhythmias in pediatric subjects. Following approval for EV ICD implantation at our center, five consecutive adolescent patients underwent implantation over a 7-month period. Patient characteristics, qualification for EV ICD placement, nuances in EV ICD implantation in each patient, and post-implant follow-up were reviewed. A total of six out of eight consecutive ICD candidates were identified as potential EV ICD candidates, with five patients undergoing eventual EV ICD implantation (average age, 16.8 years [range, 14–19 years]; four males). Indications for ICD included genetic arrhythmias (n = 3) and hypertrophic cardiomyopathy (n = 2). All patients underwent successful implantation without intraoperative or early postoperative complications. No lead dislodgement, device migration, infection, or inappropriate shocks were observed. All patients demonstrated appropriate R-wave sensing and stable lead parameters at early (4- to 6-week) and mid-term (>3- to 6-month) follow-up. Post-implant exercise testing in two patients showed no significant P- or T-wave oversensing. EV ICD implantation is feasible and well tolerated in appropriately selected pediatric patients. Most pediatric ICD referrals were candidates for EV ICD implant when integrating the EV ICD in regular practice. Implant indications differ among pediatric versus adult EV ICD recipients. While early outcomes are favorable, long-term performance and applicability in smaller patients require further investigation.

## Introduction

Implantable cardioverter-defibrillators (ICDs) play a critical role in managing pediatric patients at risk for sudden arrhythmic death. In this unique population, careful consideration of ICD type is essential to optimize safety, accommodate somatic growth, and preserve quality of life—particularly in the context of active lifestyles and long-term device management. The Aurora extravascular (EV) ICD (Medtronic, Minneapolis, MN, USA) represents a promising addition to the therapeutic toolkit for pediatric patients who meet implant criteria and seek to avoid the risks and limitations associated with transvenous, epicardial, and subcutaneous ICD systems.

While clinical experience with the EV ICD in adults is expanding, data in pediatric patients remain limited. In this case series, we present our initial experience with five consecutive pediatric patients who underwent EV ICD implantation. We highlight procedural nuances and modifications, early clinical outcomes, and device performance. We additionally propose a structured follow-up strategy and discuss the potential role of the EV ICD within the evolving landscape of pediatric defibrillator therapy.

## Methods

A retrospective review of all EV ICD implants was performed following institutional review board (IRB) approval for this case series. We identified potential candidates for the EV ICD implant among consecutive patients referred for ICD implantation over a 7-month period (July 2024 through January 2025). Patients were considered for EV ICD implantation if they met Medtronic’s inclusion criteria, which included the following: (1) an indication for primary or secondary prevention of sudden cardiac death, (2) absence of chronic pacing requirements, and (3) absence of prior sternotomy or mediastinal surgery. Eligible patients and their families were counseled on all available device options, including epicardial, transvenous, subcutaneous (S-ICD), and extravascular ICD systems. Key differences between device types were reviewed in detail. For patients who decided to undergo EV ICD implant, a backup device was also selected to be used if the EV ICD could not be successfully implanted or if defibrillation testing was unsuccessful. For families who selected the S-ICD as a backup, electrocardiogram (ECG) screening had already been performed.

This study was reviewed and approved by the Children’s Mercy Hospital Kansas City Institutional Review Board (IRB# STUDY00003513). All procedures were conducted in accordance with the ethical standards of the institution and/or national research committee and with the 1964 Declaration of Helsinki and its later amendments.

Pre-procedural planning included advanced imaging with either cardiac magnetic resonance imaging (MRI) or computed tomography, which had previously been obtained as part of an initial cardiac workup in all patient candidates. Imaging was used to assess sternal length, evaluate the relationship between the sternum and anterior pericardium, and identify any overlap of lung parenchyma. The spatial relationship between the sternum and right ventricle was assessed, and attention was given to whether right atrial tissue encroached into the retrosternal space.

Each EV ICD implantation followed Medtronic’s recommended implant sequence. All procedures were performed under general anesthesia by two electrophysiologists and a cardiothoracic surgeon in a hybrid electrophysiology laboratory equipped with biplane fluoroscopy. Both electrophysiologists completed Medtronic’s required virtual and in-person EV ICD training. The cardiothoracic surgeon assisted as needed with gaining access to and confirming entry into the retrosternal space.

Anatomic landmarks were identified by palpation and marked using a sterile skin marker, with fluoroscopic guidance used to confirm positioning. A transverse subxiphoid incision was made, and blunt dissection was performed in the plane between the rectus muscle and anterior rectus sheath, extending through the diaphragmatic attachments to enter the retrosternal space. The EV ICD lead was advanced under fluoroscopy using the dedicated Medtronic tunneling tool and secured proximally to the rectus muscle. A subcutaneous generator pocket was created in the left lateral chest wall, where the lead was tunneled and connected to the generator. In all cases, defibrillation testing was performed using an initial 25-joule (J) shock to confirm appropriate sensing and a defibrillation safety margin of at least 10 J. In the event of unsuccessful defibrillation at 25 J, a second shock at 30 J was delivered through the device with external rescue defibrillation available if needed thereafter. Patients who failed the 25-J testing underwent ventricular fibrillation re-induction and EV ICD treatment at 30 J. Following successful testing, all incisions were irrigated and closed in a standard layered fashion.

Patients remained hospitalized for overnight observation. Routine postoperative ECG, chest X-ray, and device interrogation were performed the morning after the procedure prior to discharge. Outpatient follow-up was conducted at 4–6 weeks post-implantation and then at 3- to 6-month intervals per the primary electrophysiologist’s direction. In-person follow-up included ECG and device interrogation, and remote monitoring reports were obtained every 3 months. Follow-up chest X-ray imaging was done at the discretion of the primary treating electrophysiologist. Two patients also underwent exercise stress testing with concurrent device interrogation.

## Results

Following institution and manufacturer approval to begin EV ICD implantation at our center, consecutive patients referred for ICD implantation were evaluated, with the EV ICD offered as one of four ICD system types available for implantation. A total of eight patients were referred during the 7-month period following EV ICD availability. Of these, six met inclusion criteria for the EV ICD (75%). All six families elected to pursue EV ICD implantation, with one unable to be implanted due to insurance denial and subsequently undergoing S-ICD implant instead. The remaining two ICD candidates were excluded from EV ICD consideration due to previous sternotomy.

Patient demographics and clinical histories of EV ICD recipients are summarized in **Table 1**. Most patients were male (4/5, 80%), with an average patient age of 16.8 years (14–19 years). The average height was 180 cm, and the average weight was 98.3 kg. All patients were either overweight or obese based on their body mass index (average, 30 kg/m^2^ [range, 25.4–38.8 kg/m^2^]). Primary prevention ICDs were implanted in three out of five patients. Underlying cardiac conditions included genetic arrhythmia disorders (n = 3) and hypertrophic cardiomyopathy (n = 2). Both patients with hypertrophic cardiomyopathy received primary prevention devices. Patient #1 had previously undergone S-ICD implant and subsequently experienced recurrent inappropriate shocks due to poor R-wave sensing and T-wave oversensing. While this patient originally passed S-ICD screening (single vector), significant changes in sensing followed shortly after S-ICD implant, resulting in inappropriate shocks that could not be mitigated despite exhausting all available programming options. Patient #5 was originally offered an S-ICD but failed ECG screening and subsequently wished to pursue EV ICD implantation.

**Table 1:** Patient Demographics and Clinical Summary

	Sex	Age (Years)	Height (cm)	Weight (kg)	Diagnosis	Prior Cardiac Intervention	Clinical Presentation	Primary or Secondary Prevention ICD
Case 1	Male	14	187.3	100.5	TNNI3K mutation	Electrophysiology study with ablation of two ectopic atrial foci Implantation of an implantable loop recorder Implantation of an S-ICD	Unexplained syncope with exertion, documented polymorphic ventricular tachycardia with near-syncope	Secondary
Case 2	Female	15	179	92.3	Long QT syndrome	None	Sudden cardiac arrest with prompt rescue	Secondary
Case 3	Male	19	185.4	133.5	Hypertrophic cardiomyopathy	None	Severe septal hypertrophy with significant fibrosis by MRI	Primary
Case 4	Male	17	169.1	81.7	Hypertrophic cardiomyopathy	None	Severe septal hypertrophy with significant fibrosis by MRI	Primary
Case 5	Male	19	181.3	83.4	Brugada syndrome	None	High-risk phenotype features	Primary

*Abbreviations:* MRI, magnetic resonance imaging; S-ICD, subcutaneous implantable cardioverter-defibrillator.

All five patients underwent successful EV ICD implantation without intraoperative or early postoperative complications. For patient #1 with a preexisting S-ICD, the S-ICD electrode was removed by simple traction, and the lateral chest pocket was reused for the EV ICD without the need for plication. For patient #5, the right and left lung parenchyma were in very close contact in the retrosternal space on cardiac/chest MRI. To avoid potential pleural space entry during electrode insertion, retrosternal tunneling was performed during ventilator-controlled end-expiratory breath hold. Case-specific sensing, testing, and implantation details are summarized in **Table 2**. Appropriate R-wave sensing was achieved in all patients (average, 2.8 mV [range, 2.1-4.7 mV]). No significant difference in sensing amplitude was noted based on underlying diagnosis. There was no evidence of significant P- or T-wave oversensing, and all patients demonstrated appropriate lead impedances. Four patients were successfully defibrillated with first-shock delivery of 25 J. One patient (with hypertrophic cardiomyopathy) failed with the 25-J delivery but converted with 30-J shocks. No repositioning of the electrode or generator was necessary in any case.

**Table 2:** Intraoperative Device Interrogation Data

	Sensing	Impedances	Defibrillation	Fluoroscopy
Case 1	R-wave sensing: 2.6 mV	Lead impedance: 247 Ω High-voltage impedance: 47 Ω	25J successful Shock impedance: 56 Ω	Anteroposterior view: 2 min Lateral view: 1.6 min
Case 2	R-wave sensing: 2.3 mV	Lead impedance: 247 Ω High-voltage impedance: 50 Ω	25J successful Shock impedance: 57 Ω	Anteroposterior view: 3.5 min Lateral view: 3 min
Case 3	R-wave sensing: 2.4 mV	Lead impedance: 399 Ω High-voltage impedance: 69 Ω	30J successful Shock impedance: 70 Ω	Anteroposterior view: 2.6 min Lateral view: 2 min
Case 4	R-wave sensing: 4.7 mV	Lead impedance: 285 Ω High-voltage impedance: 47 Ω	25J successful Shock impedance: 56 Ω	Anteroposterior view: 4.2 min Lateral view: 2.6 min
Case 5	R-wave sensing: 2.1 mV	Lead impedance: 360 Ω High-voltage impedance: 61 Ω	25J successful Shock impedance: 56 Ω	Anteroposterior view: 1.5 min Lateral view: 1.1 min

Note that all R-wave sensing and lead impedances were obtained from ring 1 to ring 2.

Several incidents and findings in early cases drove procedural modifications that were implemented in subsequent cases. The first modification was the use of a long, curved Kelly forceps to access the retrosternal space. As per Medtronic’s recommendation, non-instrument digital dissection is favored during retrosternal access. However, particularly in severely obese patients, digital dissection to and through the retrosternal diaphragmatic attachments is limited by finger length and thickness of the subcutaneous adipose tissue layer. With the aid of our assisting cardiothoracic surgeon, a long-curved Kelly forceps was advanced cranially along the anterior rectus with the tip pointing upward along the underside of the xyphoid and sternum. The instrument was then gently advanced through the diaphragmatic attachments to facilitate safe entry into the retrosternal space. This technique was used successfully in four out of five patients.

The second modification involved anchoring suture placement. As per Medtronic recommendations, two anchoring sutures were to be placed on the rectus muscle prior to retrosternal lead placement. In our first two implant procedures, owing to the electrode’s stiffness in comparison to transvenous and S-ICD leads, we found it difficult to predict and then fit the electrode and suture sleeve into the predetermined anchoring sites with pre-laid sutures. For subsequent cases, rather than placing two anchoring sutures to the rectus muscle before retrosternal electrode placement, only one suture was placed. This suture was placed with the knot oriented at an approximately 45° angle relative to the transverse incision. Following retrosternal electrode placement, the pre-laid anchoring suture was tied around the most proximal notch on the suture sleeve, followed by constrictor knot placement. The final anchoring suture was then placed in the rectus muscle based on the natural orientation of the lead in relation to the rectus muscle. Using this approach, lead anchoring was significantly simplified, especially in obese patients, without having to force lead anchoring into a predefined orientation as would happen if both anchoring sutures had been laid before electrode placement. This technique was employed in three out of five patients.

**Figure 1:**
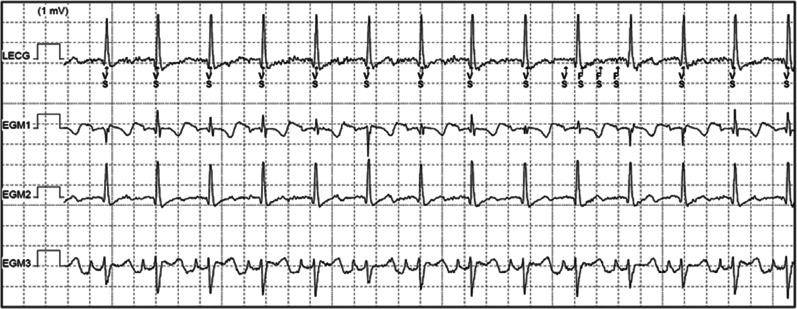
Electrograms obtained during exercise stress testing. Trivial P- and T-wave oversensing.

All patients returned for follow-up 4–6 weeks post-implantation, then at 3- to 6-month intervals thereafter. Device interrogation showed stable R-wave sensing, trivial P- or T-wave oversensing, and no inappropriate shocks. Follow-up chest X-ray was obtained in four patients demonstrating stable electrode and generator position. Two patients underwent device interrogation with exercise stress testing to evaluate sensing parameters under exercise conditions. No significant oversensing during exertion was observed **(Figure 1)**. Post-procedural testing is summarized in **Table 3**.

**Table 3:** Follow-up Device Interrogation Data

	Length of Follow-up	Sensing	Impedances	Migration on Chest X-ray	Exercise Stress Testing	Signs of Infection
Case 1	9 months	R-wave sensing: 4.4 mV	Lead impedance: 323 Ω High-voltage impedance: 71 Ω	None	Yes—no evidence of significant P/T oversensing. Appropriate R-wave sensing.	None
Case 2	8 months	R-wave sensing: 2.2 mV	Lead impedance: 371 Ω High-voltage impedance: 63 Ω	None	Yes—no evidence of significant P/T oversensing. Appropriate R-wave sensing	None
Case 3	8 months	R-wave sensing: 2.9 mV	Lead impedance: 539 Ω High-voltage impedance: 78 Ω	None	No	None
Case 4	5 months	R-wave sensing: 3.7 mV	Lead impedance: 399 Ω High-voltage impedance: 68 Ω	None	No	None
Case 5	5 months	R-wave sensing: 3.3 mV	Lead impedance: 430 Ω High-voltage impedance: 69 Ω	Not obtained	No	None

Note that all R-wave sensing and lead impedances were obtained from ring 1 to ring 2.

## Discussion

This case series presents our center’s initial experience with the Aurora EV ICD in pediatric patients. All five included patients successfully underwent device implantation without intraoperative or early postoperative complications. Defibrillation testing was successful in all cases, and short- to mid-term follow-up demonstrates stable sensing parameters, an absence of inappropriate shocks, and an absence of device migration or infection. These results support the EV ICD as a feasible and well-tolerated option in appropriately selected pediatric patients.

The increasing use of ICDs in younger populations reflects the growing recognition of their importance in preventing sudden cardiac death.^1^ Selection of an ICD system in the pediatric population requires careful consideration of body habitus, venous anatomy, underlying cardiac substrate, and psychosocial factors. Traditional transvenous ICD systems, while effective, introduce long-term risks such as venous thrombosis and occlusion, endovascular infection, and endocarditis. Patients also incur additional risks if undergoing transvenous lead extraction. Epicardial systems avoid the vasculature but require a sternotomy or thoracotomy for lead placement, are associated with longer recovery times, and may be limited by reduced system longevity.^2^ The S-ICD provides a less invasive, non-transvenous alternative, but its larger generator size, lack of pacing capability, and limited programmability, especially to address sensing issues, limit its appeal in younger patients.^1,3^ A comparison of ICD types for pediatric patients is presented in **Table 4**.

**Table 4:** Comparison of Implantable Cardioverter-defibrillator Systems^1,3–9^

	Epicardial ICD	Transvenous ICD	Subcutaneous ICD	Extravascular ICD
Lead Placement	Epicardial (heart surface)	Intravenous (endocardial)	Subcutaneous (parasternal)	Retrosternal (mediastinal)
Typical Patient Size	Suitable for smaller patients, including <30 kg	≥30–35 kg (depending on vascular access suitability)	≥40–50 kg	≥40–50 kg
Vascular Access Required	No	Yes	No	No
Pre-implant Screening	No	No	Yes	Yes (advanced imagining)
Pacing Capability	Full pacing capability, including chronic pacing, and cardiac resynchronization therapy	Full pacing capability, including chronic pacing, anti-tachycardia pacing, and cardiac resynchronization therapy	Post-shock pacing	Anti-tachycardia pacing, post-shock pacing, and pause-prevention pacing
Longevity of Device	5–8 years (varies based on pacing and defibrillation use)	10–12 years (varies based on pacing and defibrillation use)	6.3–7.3 years (based on defibrillation use)	10+ years (pending long-term data)
Approximate Device Volume	30–35 cm^3^	30–35 cm^3^	59.5 cm^3^	33 cm^3^
Potential Complications	Lead fracture and dislodgement Device infection Pericarditis Pocket hematoma Migration	Lead fracture and dislodgement Venous thrombosis, occlusion, and stenosis Device infection and endocarditis. Vascular injury Pocket hematoma Migration	Lead fracture and dislodgement Device infection Pocket hematoma Migration	Lead fracture and dislodgement Device infection Pocket hematoma Migration
Activity Restrictions	Moderate restriction to avoid direct chest and abdominal trauma	Strict restrictions on contact sports; restriction from repetitive upper-extremity motions	Minimal restrictions Avoid direct injury to ICD	Minimal restrictions Avoid direct injury to ICD

*Abbreviation:* ICD, implantable cardioverter-defibrillator.

The EV ICD fills a critical niche by combining the non-transvenous benefits of the S-ICD with additional advantages: a smaller generator, anti-tachycardia pacing (ATP) functionality, and enhanced programmability. In our experience, patients and families cited generator size, ATP availability, and lifestyle compatibility as key factors influencing their decision to pursue EV ICD implantation.

Retrosternal sensing carries the potential to alleviate sensing failures that may preclude S-ICD candidacy in some patients or result in inappropriate shocks following S-ICD implantation. Interestingly, despite issues with T-wave oversensing and fluctuating R-wave amplitudes with the S-ICD in patient #1 in our cohort, no such issues were observed with EV ICD sensing. Additionally, patient #5 has done extremely well following EV ICD implant without any sensing concerns, despite failure to pass S-ICD ECG screening. Our experience underscores the EV ICD’s potential role in patients with challenging sensing profiles.

Our experience suggests broad applicability of the EV ICD in adolescents with adult-sized habitus. Of eight patients referred for ICD implantation over the study period, six met EV ICD inclusion criteria and five ultimately underwent implantation. All EV ICD candidates would have undergone implant barring insurance denial in one patient. The spectrum of underlying diagnoses included both genetic arrhythmia conditions and hypertrophic cardiomyopathy, illustrating the device’s versatility across common pediatric arrhythmic substrates. Most implants were for primary prevention, reflecting growing adoption of proactive risk-reduction strategies in pediatrics.^4^ Notably, substrate selection in pediatrics is different from adults, with the EV ICD pivotal study demonstrating that most adults in that trial suffered from non-ischemic or ischemic cardiomyopathy with left ventricular systolic dysfunction.^5,6^

**Figure 2:**
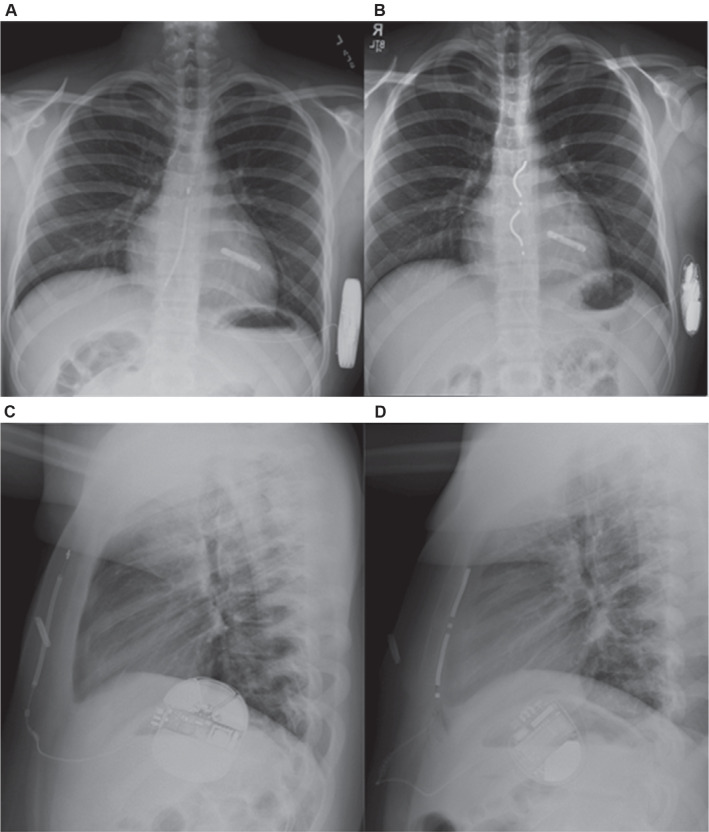
Transition from a subcutaneous implantable cardioverter-defibrillator (ICD) (S-ICD) to an extravascular ICD (EV ICD) without pocket plication (patient #1). The previously implanted S-ICD system **(A, C)** was explanted, and an EV ICD was successfully implanted in its place **(B, D)**. The original S-ICD generator pocket was reused without the need for plication, and post-implant and follow-up imaging demonstrated stable generator positioning without evidence of migration. The EV ICD pulse generator was securely anchored to the serratus anterior muscle using the device-integrated anchoring eyelets. An implantable loop recorder is also seen in all the images.

Procedurally, we implemented two modifications that may facilitate EV ICD implantation in young patients. First, the use of a long, curved Kelly forceps enabled safe and controlled access to the retrosternal space in patients, particularly in those with increased subcutaneous adiposity, where digital dissection alone was limited by digital reach. Second, pre-placement of a single angled anchoring suture provided greater flexibility in lead positioning during tunneling, improving electrode alignment and tension distribution. For our one patient with prior S-ICD implantation, the S-ICD generator pocket was reused without the need for plication, thereby simplifying the procedure and reducing tissue disruption **(Figure 2)**.

Our initial EV ICD implant experience also provides insights into cardiothoracic surgical support and participation during the procedure. A cardiothoracic surgeon directly assisted with substernal access during our first three cases but only provided confirmation of substernal entry during the subsequent two implants. Surgical participation during our initial cases also led to refinements in implant technique as well. Beyond the first five implant procedures, direct cardiothoracic surgical involvement is not required, and the procedure can be reasonably and safely performed by electrophysiologists, as is the case at our center. Beyond the manufacturer’s recommendations during early implant experience, the individual implanter’s experience, level of comfort with the procedural elements, and institutional resources should guide the degree of surgical involvement during EV ICD implants.

Routine post-implantation exercise stress testing was performed in two patients and revealed no significant concerns for P- or T-wave oversensing, even at maximal exertion. We advocate for this testing in active pediatric patients, particularly those with a history of oversensing or high-risk activity profiles. It offers a valuable opportunity to confirm sensing integrity in real time by leveraging device-based tools for sensing optimization—such as oversense prevention algorithms, programmable blanking periods, decay delay, adjustable sensitivity thresholds, and sensing vector selection—all of which work collectively to reduce the likelihood of inappropriate therapies.

Despite encouraging early outcomes, several limitations warrant discussion. Our patient cohort consisted entirely of adolescents with adult-sized thoracic anatomy, which limits generalizability to younger or smaller children. While we would have considered EV ICD implant in younger and smaller ICD candidates, no such candidates were among the consecutive patients referred for ICD implantation during our initial 7-month period of EV ICD availability. While successful EV ICD implantation has been reported in a patient as young as 2 years old,^10^ the feasibility of EV ICD implantation in smaller patients is constrained by factors such as reduced sternal length, narrower retrosternal space, and greater likelihood of atrial tissue overlap. These anatomical limitations may increase the risk of inappropriate sensing, particularly P-wave oversensing, if the distal electrode segment overlaps the right atrium. Additionally, as children grow, the fixed anchoring of the electrode at the level of the diaphragm raises the possibility of functional “downward migration” of the lead relative to the heart, which may compromise ventricular sensing and defibrillation efficacy over time. The impact of somatic growth on lead performance, stability, and sensing vectors remains unknown and may represent a unique long-term challenge in pediatric recipients. Another key consideration is lead extractability. The retrosternal electrode course and potential for fibrotic encapsulation near the diaphragm or sternal undersurface may make EV ICD electrode removal technically difficult in cases of infection or lead malfunction.

Our implant experience indicates suitability of the EV ICD in adolescent patients, where body size and habitus are near or equal to adult dimensions. However, limited experience and outcomes in younger and smaller pediatric patients prevents determination of a clear minimum height and weight threshold below which EV ICD implant should be discouraged. Present experience and limited long-term pediatric outcomes data highlight the need for cautious patient selection and ongoing study before more liberalized EV ICD use in younger and smaller patients. Candidacy for the EV ICD in pediatric patients should prudently factor in anatomical considerations, growth potential, and family-centered decision-making, recognizing that the risk–benefit profile may vary substantially between individuals.

Encouragingly, early reports are beginning to demonstrate the potential feasibility in even younger children. A recent case by Rabin et al. described the successful implantation of an EV ICD in a 2-year-old boy with Brugada syndrome, following a witnessed out-of-hospital cardiac arrest. The procedure used a minimally invasive surgical approach, with small incisions and substernal tunneling tailored to the patient’s anatomy. The patient’s postoperative course was uncomplicated, and the device successfully delivered therapy for a subsequent arrhythmic event. This case provides proof of concept that, under carefully selected circumstances, EV ICD implantation may be achievable in patients well below the typical size threshold.^10^ While such cases remain exceptional, they offer a glimpse into the evolving potential of this technology as experience and comfort with the system grow. Our findings, paired with emerging reports, suggest that the procedural strategies and anatomical considerations outlined here may serve as a foundation for responsible expansion into younger patient populations.

## Conclusion

Our initial experience with the EV ICD in young and active patients has been highly encouraging, demonstrating its potential and broad applicability in the pediatric population. In our experience, patients and families consistently favored the EV ICD for its smaller size, avoidance of vascular and endocardial risks, and ability to support unrestricted mobility of the upper extremities, outweighing concerns about the device’s novelty and limited long-term data. Medtronic implantation guidelines, coupled with our minor modifications, have resulted in safe EV ICD implantation and enhanced procedural efficiency. While uncertainties regarding somatic growth, lead management, and long-term outcomes remain, the EV ICD addresses many critical needs of younger patients at risk for sudden cardiac death. Continued experience and data collection will be vital to refining its use and expanding its role in pediatric care.
