# Erratum: Short- and Mid-term Outcomes of the Extravascular Implantable Cardioverter-defibrillator in Pediatric and Adolescent Patients: A Single-center Experience

**DOI:** 10.19102/icrm.2026.17057

**Published:** 2026-05-15

**Authors:** Nikola Dragisic, Emily R. Backes, Lindsey Malloy-Walton, Edo K. Bedzra, William J. Gibson, Philip M. Chang

**Affiliations:** 1 Ward Family Heart Center, Children’s Mercy Kansas City, Kansas City, MO, USA

The following corrections have been made to the article “Dragisic N, Backes ER, Malloy-Walton L, Bedzra EK, Gibson WJ, Chang PM. Short- and mid-term outcomes of the extravascular implantable cardioverter-defibrillator in pediatric and adolescent patients: a single-center experience*. J Innov Cardiac Rhythm Manage.* 2025;16(11):6488–6495. doi: 10.19102/icrm.2025.16113.”

## Error 1

###  

Table 4 has been updated to include the corrected Aurora extravascular (EV) implantable cardioverter-defibrillator (ICD) (Medtronic, Minneapolis, MN, USA) volume, along with the appropriate post-shock pacing and pause prevention features.

The corresponding author explains that the incorrect values listed in the published table “[were] an error on [their] end that occurred while revising the table during multiple rounds of edits.” To facilitate this correction, the manuals for all currently available ICD systems were reviewed again to ensure all information in the table reflects “current devices and not older technology.” The SICD manual has also been added to the reference list as “the most relevant EV ICD competitor”, in the author’s opinion.

#### Correction

The table is now correct as presented.

## Error 2

###  

Elements of the reference list were incorrect; parts of some references (eg, author names, journal names) were inappropriately mixed together, and titles were often incorrect.

The corresponding author expresses that there was never any intention to misrepresent the literature; instead, this error resulted from a workflow failure involving bibliographic software use and multiple rounds of edits.

#### Correction

The reference list is now correct as presented.

##### Final Word

The corresponding author expresses that they sincerely apologize for these errors and take full ownership and responsibility.

Both corrections have been made to the online version of the article.

